# Comparison of the accuracy of different impression procedures in case of multiple and angulated implants: Accuracy of impressions in multiple and angulated implants

**DOI:** 10.1186/s13005-020-00225-3

**Published:** 2020-05-04

**Authors:** M. Wafa Richi, Sevcan Kurtulmus-Yilmaz, Oguz Ozan

**Affiliations:** Department of Prosthodontics, Near East University Faculty of Dentistry, Lefkosa, Mersin10, Ankara, Turkey

## Abstract

**Background:**

There is no consensus in the literature regarding the impression procedures in the presence of multiple and angulated implants.

**Methods:**

Three maxillary master models with 6 implants bilaterally positioned in anterior, premolar and molar regions were fabricated. In model 1, all implants were placed in parallel; in models 2 and 3, anterior implants were buccally inclined and posterior implants were distally inclined in 10- and 20-degrees, respectively. Three different impression copings (hexed, non-hex, multi-unit) and two different impression techniques (splinting and non-splinting) were tested. A total of 180 impressions (*n* = 10 per group) were made using mono-phase vinyl poly-siloxane. Master models and duplicate casts were scanned by a 5-axis laboratory scanner and data were transferred to a software program for the alignment of master and duplicate copings. Coronal and angular deviations were calculated, and data were statistically analyzed.

**Results:**

For angulated models, the lowest deviation values were detected at the splinted non-hex coping group (*P* < 0.05).

**Conclusions:**

Implant angulation, impression coping type, and splinting the impression copings had significant effects on the accuracy of impressions.

**Trial registration:**

Not applicable.

## Introduction

Advancements in implant technology, recent developments in techniques and materials, and long-term success of the implants led implant-supported prostheses to become the most preferred treatment option for the functional and aesthetic rehabilitation of edentulism. The passive fit of implant-supported prostheses is one of the most important factors affecting the success of treatment protocol. In cases where passive fit cannot be achieved between the implant components and the prosthetic superstructure, many biological and mechanical complications such as screw loosening or fracture, increased plaque accumulation, loss of osseointegration or fracture of the implant may be encountered [1]. The first step in ensuring the passive fit is to make an accurate impression and to transfer the 3-dimentional positions of implants into the laboratory models. Impression technique, impression material [2], splinting / non- splinting of impression copings, splint material, number and angle of the implants [3] are the factors that affect the accuracy of the impression.

Due to the anatomical and aesthetic limitations, it is not always possible to place the implants parallel to each other. It has been stated that in the presence of 4–6 implants, the impressions made from parallel implants show higher accuracy than the ones made from angulated implants [4–6]. In the case where multiple implants are available and an angular difference of more than 15° exists between implants or impression copings, the use of open-tray (direct) impression technique and splinting of impression copings are recommended [7, 8]. The most preferred impression materials in implant dentistry are polyether and vinyl poly-siloxane [7, 9]. The impression material used in the open-tray impression technique must show sufficient rigidity to maintain the position of the impression coping and prevent it from being displaced during removal from the mouth. Splinting the impression copings is recommended in order to increase the accuracy of impression and avoid the distortion of impression material, particularly while fastening the implant analogs to their respective copings [10, 11]. Recent studies have reported that in the case of complete edentulism rehabilitated with 4 or more implants, splinted impression techniques provided more accurate impression in comparison to non-splinted techniques [12–15].

Implant-abutment connections are of two types; internal and external. Currently, internally connected implants are widely used. A study [16] that examined the effect of internal and external connections on the accuracy of the impression concluded that, if the implants were parallel, the connection type did not affect the accuracy of the impression; however, it has also been reported that the accuracies of impressions obtained from internally connected implants significantly reduced when there were angular differences between impression copings of corresponding implants [4, 16]. The connection area of the internally connected implants is longer and wider, compared to externally-connected ones. In the case of angulated implants, longer and wider connection area may cause displacement of the impression copings while removing impression copings from the mouth and may increase the amount of distortion [17]. When the open tray impression technique is used in implants with internal connection and the impression copings are splinted with a rigid material, distortion risk caused by the connection area increases during removal. To circumvent this risk, non-hex impression copings have been developed. In ‘non-hex’ copings, unlike conventional ‘hexed’ impression copings, there is a shallow connection area that fits into the implant. Manufacturers recommend the use of non-hex copings when multiple implants are used, angular difference presents between the implants, and the copings are splinted [18].

In implant dentistry, the impression can be made in implant or abutment level. Implant level technique has some advantages over abutment level technique since it permits the selection and customization of the abutment in the laboratory [19]. However, especially in complete edentulism cases, when there are multiple and angulated implants and screw-retained restorations are planned, multi-unit abutments are preferred. Multi-unit abutments not only manage the angulation differences, but also eliminate the direct connection between impression copings and implants, and thereby reduce the deformation risk of impression material during removal [20]. Previous studies [21, 22] including 4 implants highlighted that abutment-level impressions give better results in terms of accuracy when angulated implants exist. However, the data regarding the comparison of impression levels in the presence of multiple and angulated implants are very limited.

Impression level (abutment/implant), impression coping (hexed/non-hex), splinting or non-splinting the copings, and angular differences among impression copings are the factors that may affect the accuracy of impression and thereby, precise fit of the prosthesis in the presence of multiple implants. Therefore, the aim of this study was to investigate the effects of these variables on the accuracy of impression via digital evaluation methods in the case of 6 implants placed in the edentulous maxillary arch. The null hypotheses of this study were that (1) implant angulation, (2) impression coping type, and (3) splinting or non-splinting copings would not affect the accuracy of impressions.

## Materials and methods

**Fig. 1:**
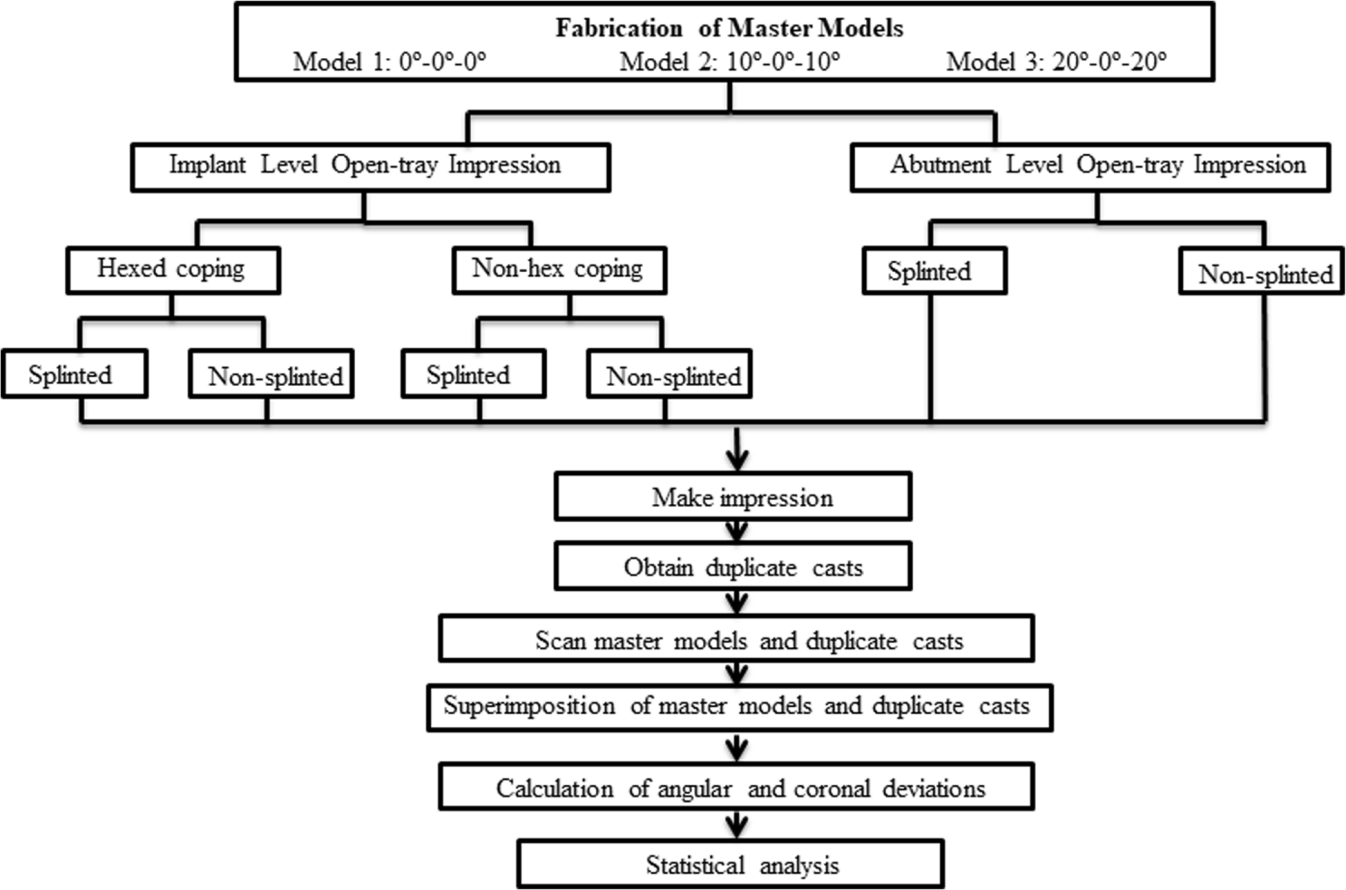
Flowchart of the study design

**Fig. 2:**
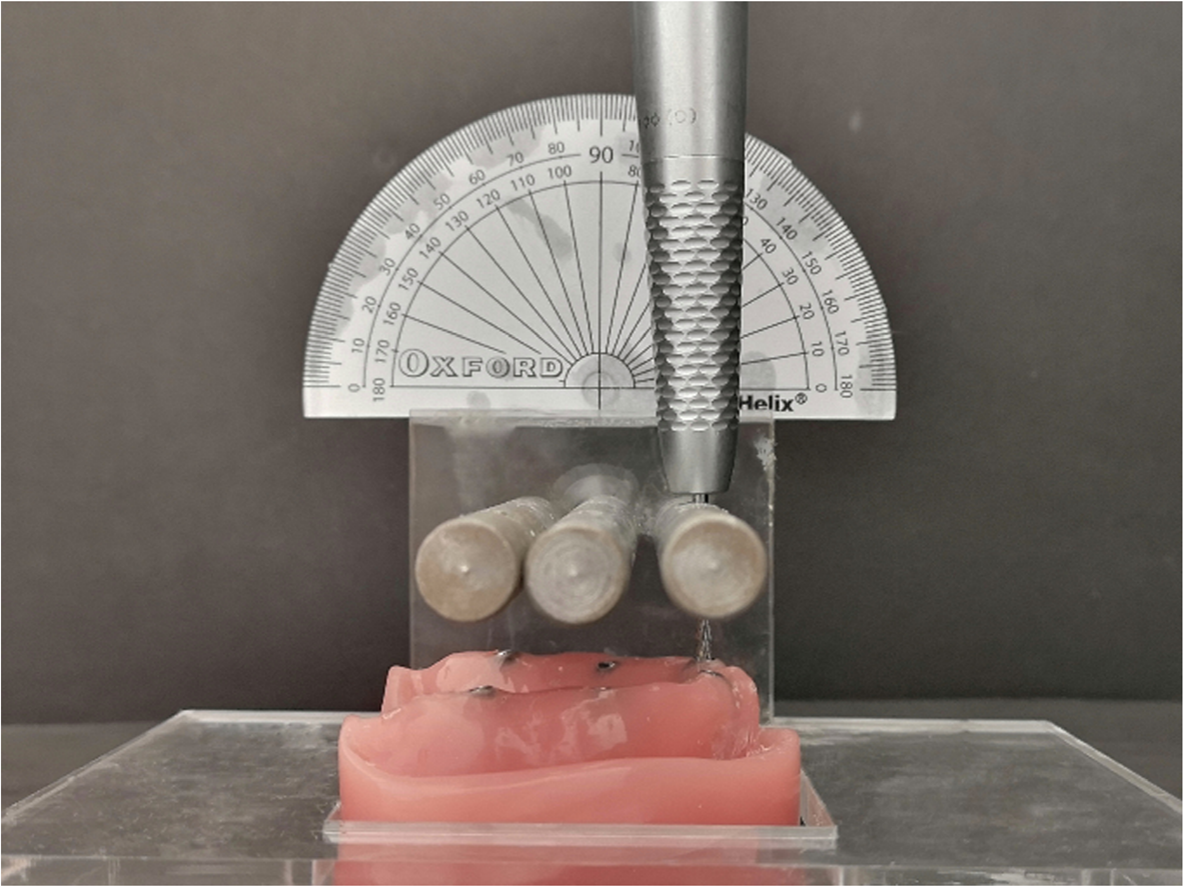
An appliance was designed and fabricated to place the implants in different angulations

**Fig. 3:**
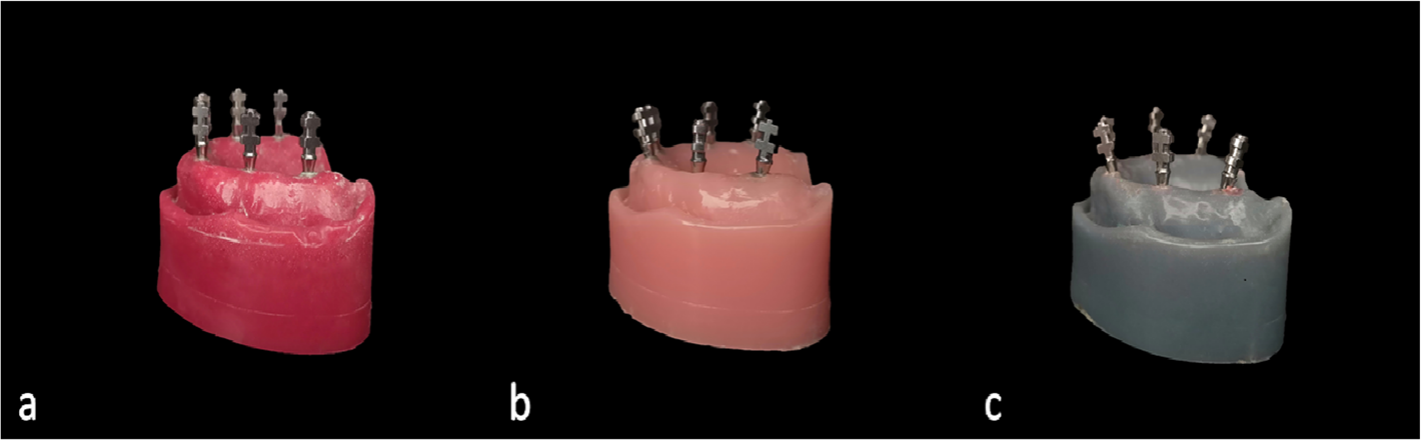
Master models with (**a**) parallel, (**b**) 10-degrees angulated, (**c**) 20-degrees angulated copings

Figure 1 describes the study design in a flow chart. Three master models were fabricated by pouring acrylic resin (Pegasus Plus Repair Acrylic; Davis Schottlander & Davis Ltd., Hertfordshire, England) into the silicon matrixes (AG-3; Frasaco GmbH, Greenville, USA) to simulate edentulous maxillary arch. A specific appliance was aimed to use for standardization of the locations and angulations of implants. Thus, to achieve standard tessellation language (STL) data, 3 master models were scanned by using a laboratory scanner (inEOS X5; Dentsply Sirona, Charlotte, North Carolina, USA). In accordance with the STL data of the jaw model, a specific appliance with metal holes which were created for placement of the demo implants (T6 4110; NucleOSS, Menderes, İzmir, Turkey) at 3 different angulations was designed by using a 3D design program (Rhinoceros; McNeel Europe, Barcelona, Spain). With the guidance of the metal holes, implant sockets were prepared at different angulations by using a rotary instrument (Fig. 2). Then, implants were fixed into the master casts with auto-polymerizing acrylic resin (HinriPress, Gosler, Germany). Each master model had 6 implants in the lateral, first premolar and second molar regions, bilaterally. Depending on the angulation of the implants, three different master models were obtained as follows:

- Model 1: All implants were placed in perpendicular to horizontal plane, parallel to both vertical axis and each other (Fig. 3a).

- Model 2: Implants in the lateral region were mesiobuccally inclined 10° to the vertical axis; implants in the premolar region were placed in perpendicular to horizontal plane and parallel to the vertical axis; implants in the molar region were distally inclined 10° to the vertical axis (Fig. 3b).

- Model 3: Implants in the lateral region were mesiobuccally inclined 20° to the vertical axis; implants in the premolar region were placed in perpendicular to horizontal plane and parallel to the vertical axis; implants in the molar region were distally inclined 20° to the vertical axis (Fig. 3c).

Three different points were arranged on the master models to serve as reference points during the superimposition process. Considering the variables (impression levels, techniques, copings and the implant angulations), a total of 180 impressions were aimed to made (*n* = 10). Therefore, 60 custom impression trays were fabricated for each master model by using light polymerizing base plates (Plaque Photo, W + P Dental, Hamburg, Germany) and then polymerized (Tray Lux, Ampac Dental, Rockdale, Australia). Trays were perforated to access the coronal ends of the copings according to the principles of open-tray technique.

**Fig. 4:**
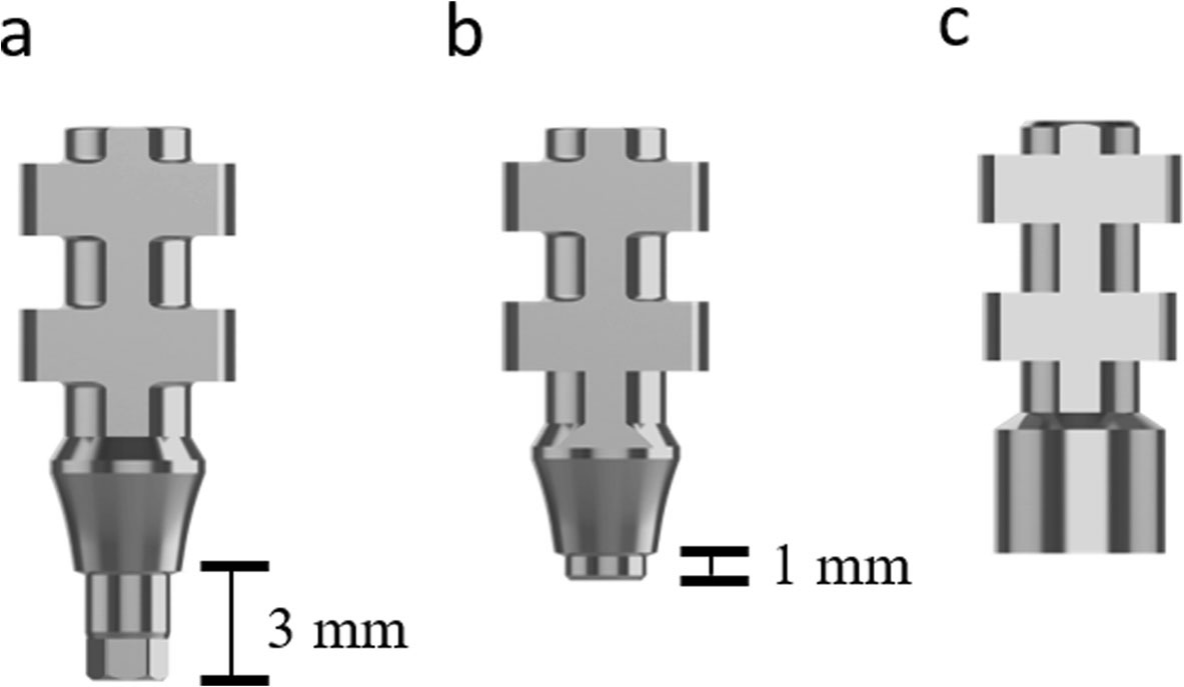
Impression copings used in the study: (**a**) hexed, (**b**) non-hex, (**c**) multi-unit impression copings

**Fig. 5:**
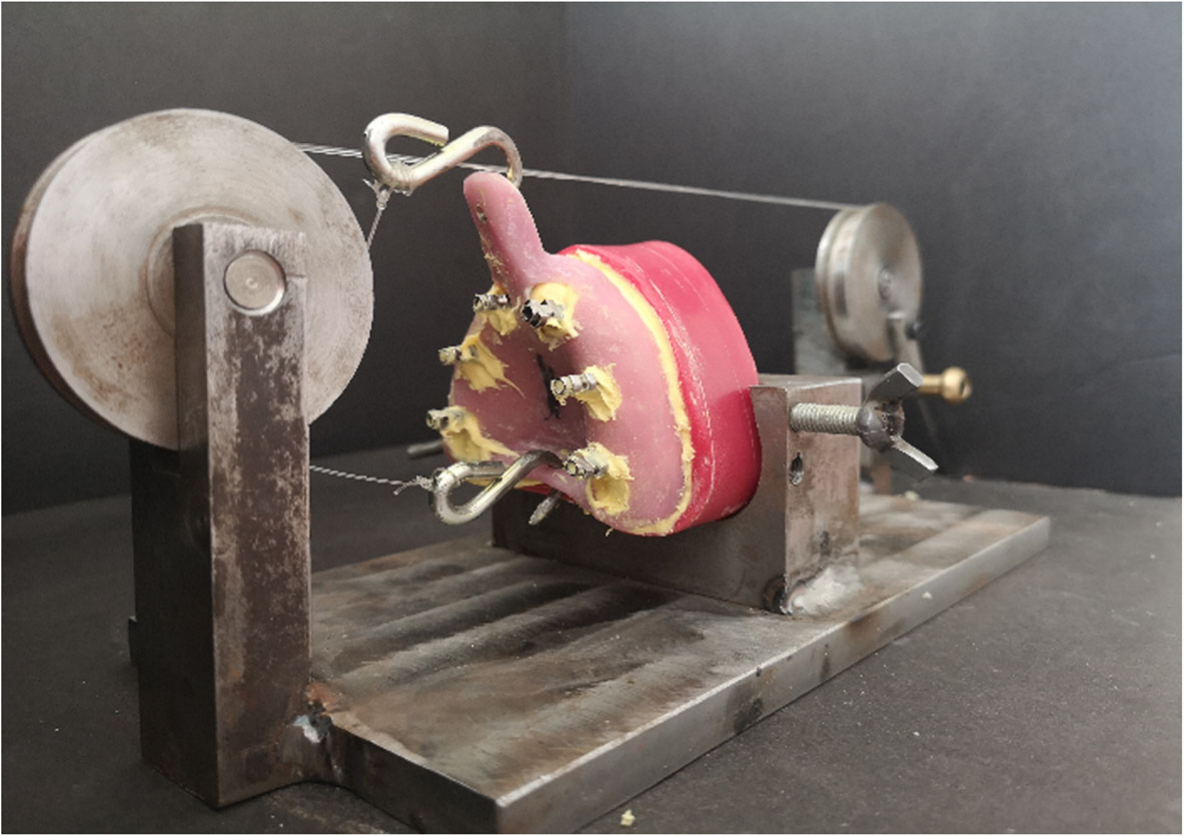
A special apparatus was fabricated to simulate the direction of tray removal from upper jaw and to standardize the removal force

All impression procedures were performed by a single calibrated clinician (M.W.R.). Before making the impression, inner surfaces of the trays were coated with tray adhesive (Universal Tray Adhesive; Zhermack, BadiaPosleine, Italy) and left to dry for 2 min following the manufacturer’s instructions. Impressions were made with a mono-phase vinyl poly-siloxane impression material (Elite HD+ Monophase; Zhermack, BadiaPosleine, Italy) that was prepared by using an automated mixing device (MixStar eMotion, DMG, Ridgefield Park, USA). All screwing procedures were done by the same clinician (M.W.R.) using a hand screwdriver. To simulate the clinical conditions, screws were tightened to the first point when solid resistance was felt. For the implant level open-tray impressions, hexed (T6 32,604; NucleOSS) (Fig. 4a) or non-hex (T6 32,605; NucleOSS) (Fig. 4b) impression copings were screwed directly to the implants. For abutment level impressions, firstly screws of multi-unit abutments (T6 32,431; NucleOSS) were tightened to the implants with a torque-wrench (15 N) and subsequently, multi-unit impression copings (T0 32,608; NucleOSS) (Fig. 4c) were attached to the abutments. For all splinted open tray technique groups, impression copings were splinted using dental floss and auto-polymerizing acrylic resin (Pattern Resin LS, GC America Inc., Alsip, IL, USA). The splint was sectioned after 24 h, and reconnected just before the impression process with the same auto-polymerizing acrylic resin. In all test groups, impression material was injected around the impression copings, and the custom tray that was loaded with impression material was placed onto the master models. A 5 kg weight was applied over the trays during polymerization of the material and material was left for 6 min for setting. To simulate removal direction of the tray from the upper jaw and standardize the removal force, an apparatus was fabricated. The apparatus consists of two pulleys, a platform to stabilize the cast model, two metal hooks and fishing line. The metal hooks were connected to the tray via two holes located at handle and post palatal region of the tray (Fig. 5). After the setting of impression material, impression copings were unscrewed and master models were fixed to the apparatus, and a removal force was applied by turning the pulley in clockwise direction until the impression was separated from the model (Fig. 5). Impression copings were screwed to the implant (T6 32,201; NucleOSS) and multi-unit (T0 32,202; NucleOSS) analogues. Type IV dental stone (Zhermack Elite Rock; Zhermack, BadiaPosleine, Italy) was poured into the impressions and after the setting of dental stone, duplicate casts were gently separated from the impressions.

**Fig. 6:**
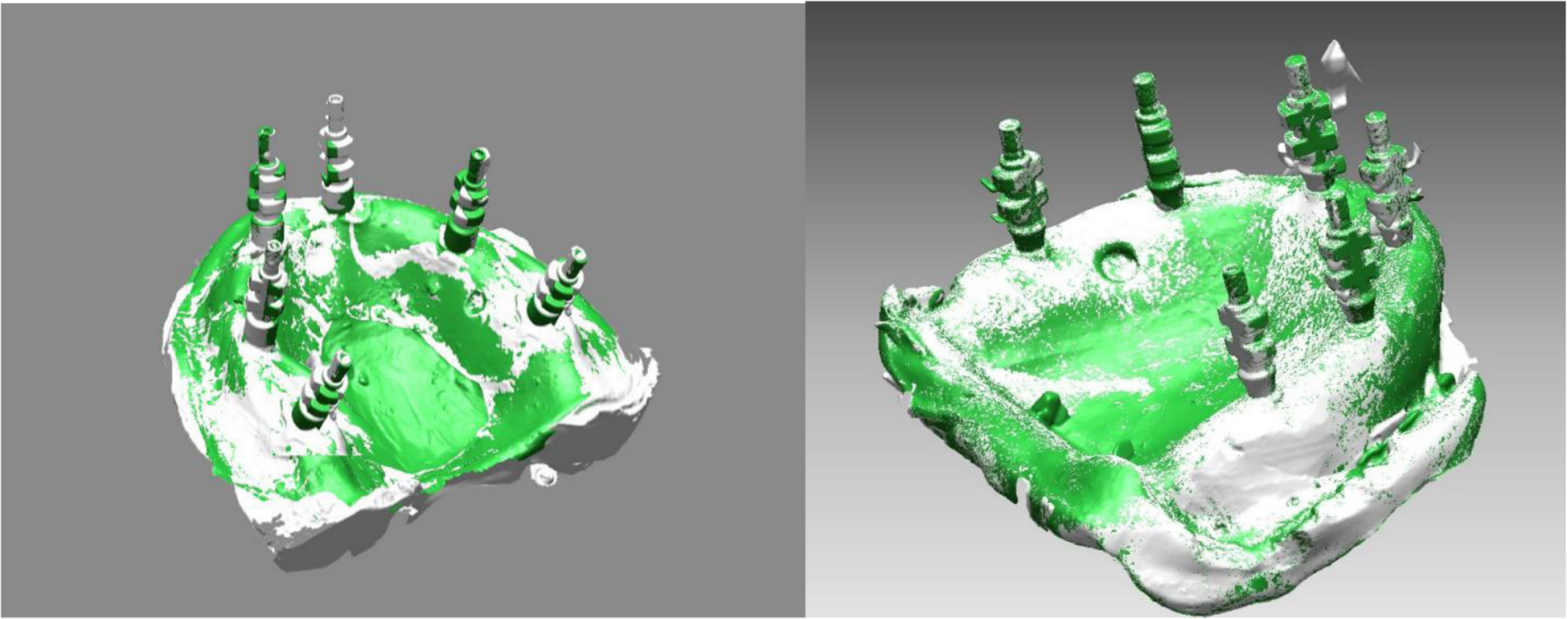
Alignment of master models and duplicate casts by superimposition of the reference points

Master models and all duplicate casts were scanned by using a 5-axis laboratory scanner (inEOS X5; Dentsply Sirona, Charlotte, North Carolina, USA) with a 2.1 μm accuracy ratio. A scanning powder (CEREC Optispray, Dentsply Sirona, Charlotte, North Carolina, USA) was applied to the master models and duplicate casts to avoid the surface reflection. The scans of duplicate casts were aligned to the scan of corresponding master model to observe the superimposition of reference points with the aid of software (VRMesh Studio, VirtualGrid Inc., Bellevue City, WA, USA) (Fig. 6).

**Fig. 7:**
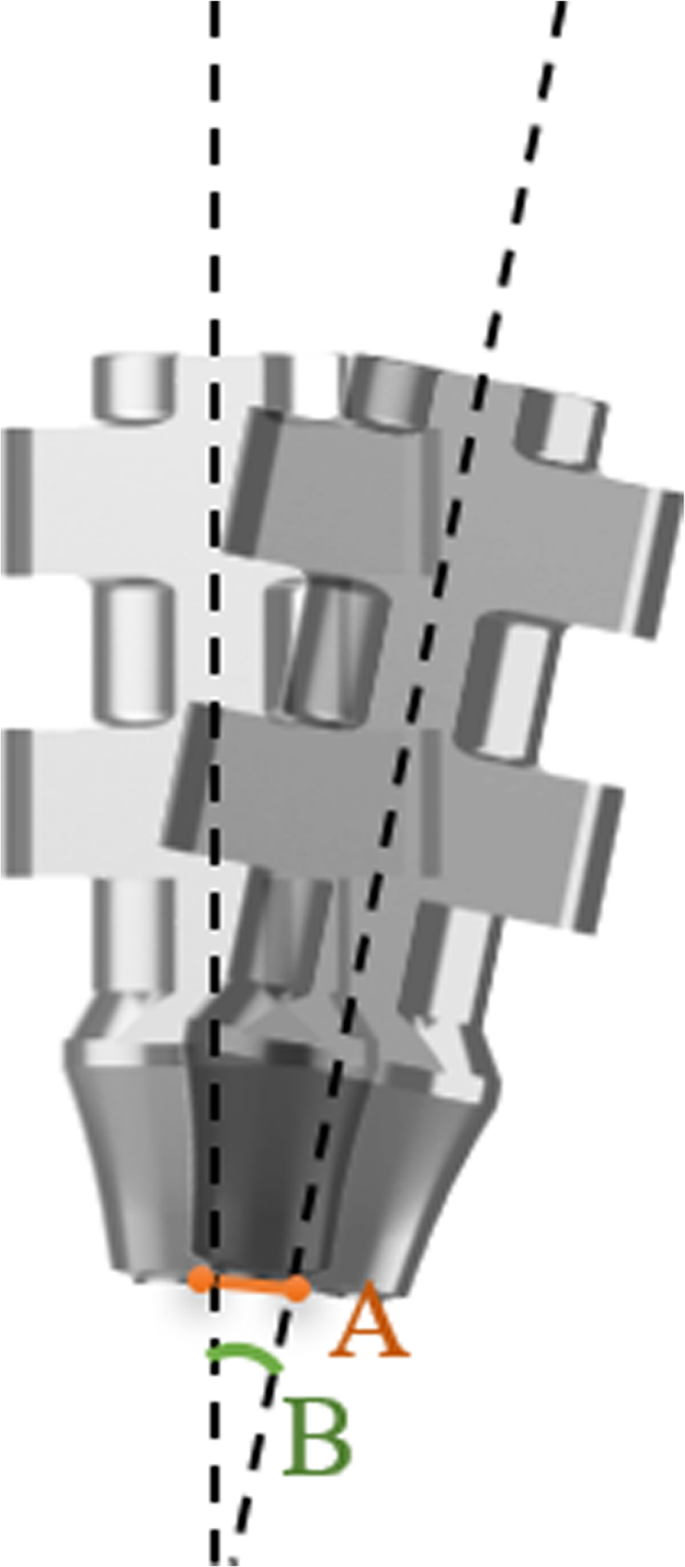
Depiction of coronal (**a**) and angular (**b**) deviations between master and duplicate copings

**Fig. 8:**
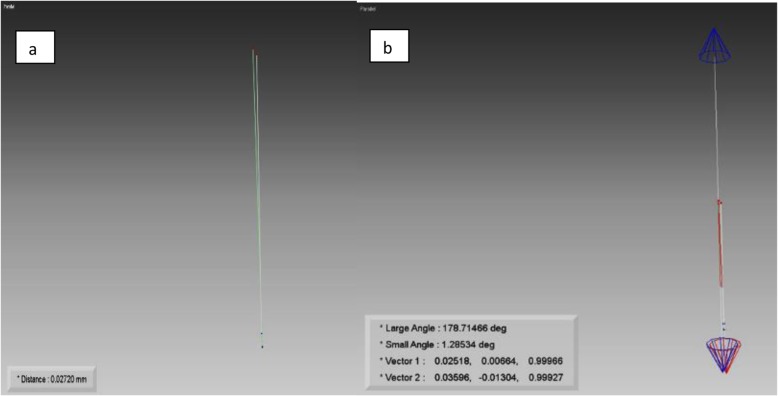
Measurement of coronal (**a**) and angular (**a**) deviations between master and duplicate copings with the use of software

Each coping on the master model and duplicate casts was converted to a cylinder by selecting two points on the long axis of the coping. One point was identified at the bottom of the coping and another point at the top of the coping by using x-, y-, z-coordinates. After alignment of the master and duplicate copings, the linear difference (in mm) between the bottom points of the copings was defined as coronal deviation and the angular difference (in degrees) detected between the long axes of copings was defined as angular deviation (Fig. 7). These deviation values were calculated by a single observer by detecting the analytical coordinates of the points (Fig. 8). Software calculated the coronal and angular deviations by using the following formulae, respectively:

$$ D=\sqrt[2]{\left({X}_{\overrightarrow{v}}-{X}_{\overrightarrow{w}}\right)+\left({Y}_{\overrightarrow{v}}-{Y}_{\overrightarrow{w}}\right)+\left({Z}_{\overrightarrow{v}}-{Z}_{\overrightarrow{w}}\right)} $$

$$ \cos \left(\theta \right)=\frac{\overrightarrow{v}\bullet \overrightarrow{w}}{\left\Vert \overrightarrow{v}\right\Vert \bullet \left\Vert \overrightarrow{w}\right\Vert } $$

where *v* values represent the coordinates on master cast and *w* values represent coordinates on duplicate cast.

Mean coronal and angular deviations of six copings on each duplicate casts were calculated, and mean deviation values for each cast were statistically evaluated. Shapiro–Wilks test was conducted to check the normality of data. Deviation values were analyzed by three-way analysis of variance (ANOVA). The considered variables were implant angulation (parallel, 10 degrees, 20 degrees), impression coping (hexed, non-hex, multi-unit) and impression technique (splinted, non-splinted). Comparisons were carried out with the aid of Bonferroni Post Hoc test when significance was detected. Values of *P* < 0.05 were accepted as statistically significant.

## Results

**Fig. 9:**
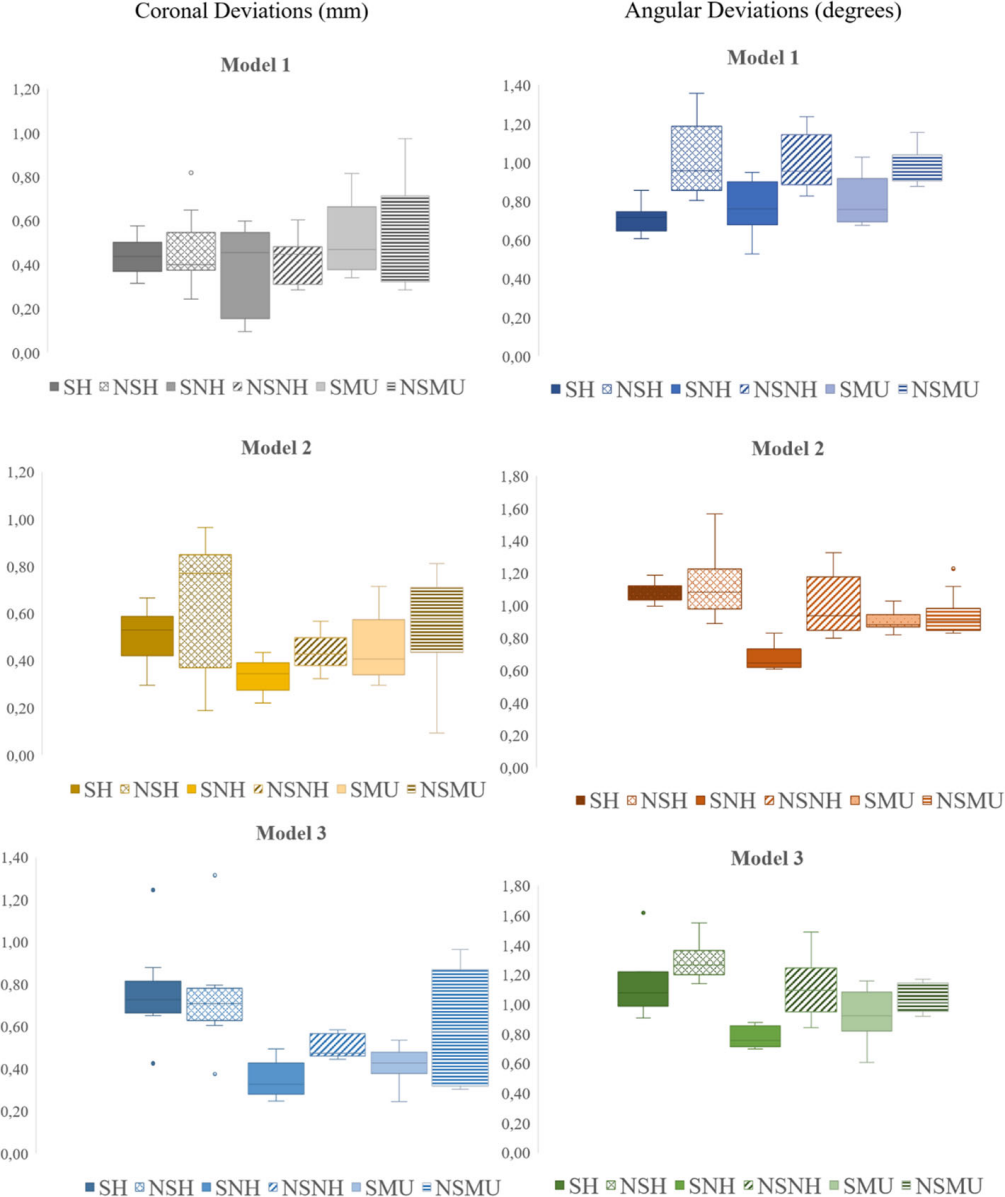
Box plot diagrams of coronal and angular deviations of duplicated casts obtained from different impression procedures (SH: Splinted-hexed coping; NSH: Non splinted-hexed coping; SNH: Splinted-non hex coping; NSNH: Non splinted-non hex coping; SMU: Splinted-multi unit coping; NSMU: Non splinted-multi unit coping)

The mean coronal and angular deviation values of impression copings with their standard deviations are presented in Tables 1 and 2, respectively. Statistical analysis revealed that implant angulation, impression technique, and impression coping type had statistically significant effects on the coronal and angular deviations of copings (Tables 3 and 4, Fig. 9).

**Table 1:** Mean ± standard deviation (SD) of coronal deviation (mm) of impression copings. Significance (Sig.) columns show the statistical groups. Same capital letters in the same row and same lower cases in the same column show no statistically significance (*P* > 0.05)

Test groups			Model 1		Model 2		Model 3	
			Mean ± SD	Sig.	Mean ± SD	Sig.	Mean ± SD	Sig.
Implant level impression techniques	Splinted hexed coping	SH	0,41 ± 0,08	A,a	0,5 ± 0,11	B,a	0,76 ± 0,23	C,a
	Non-splinted hexed coping	NSH	0,46 ± 0,13	A,a	0,62 ± 0,26	B,b	0,75 ± 0,26	C,a
	Splinted non-hex coping	SNH	0,39 ± 0,21	A,a	0,33 ± 0,04	A,c	0,35 ± 0,07	A,b
	Non-splinted non-hex coping	NSNH	0,42 ± 0,12	A,a	0,46 ± 0,08	B,d	0,5 ± 0,05	B,c
Abutment level impression techniques	Splinted multi-unit coping	SMU	0,49 ± 0,17	A,b	0,45 ± 0,11	A,d	0,42 ± 0,08	A,b
	Non-splinted multi-unit coping	NSMU	0,51 ± 0,22	A,b	0,57 ± 0,19	B,a	0,61 ± 0,27	C,d

**Table 2:** Mean ± standard deviation (SD) of angular deviation (degrees) of impression copings. Significance (Sig.) columns show the statistical groups. Same capital letters in the same row and same lower cases in the same column show no statistically significance (*P* > 0.05)

Test groups			Model 1		Model 2		Model 3	
			Mean ± SD	Sig.	Mean ± SD	Sig.	Mean ± SD	Sig.
Implant level impression techniques	Splinted hexed coping	SH	0,7 ± 0,06	A,a	1,05 ± 0,04	B,a	1,13 ± 0,20	C,a
	Non-splinted hexed coping	NSH	1,08 ± 0,21	A,b	1,19 ± 0,18	B,b	1,28 ± 0,13	C,b
	Splinted non-hex coping	SNH	0,75 ± 0,11	A,a	0,65 ± 0,07	A,c	0,77 ± 0,02	A,c
	Non-splinted non-hex coping	NSNH	1,09 ± 0,15	A,b	1,02 ± 0,18	A,a,b	1,12 ± 0,22	B,b
Abutment level impression techniques	Splinted multi-unit coping	SMU	0,84 ± 0,12	A,a	0,88 ± 0,03	A,d	0,91 ± 0,17	A,d
	Non-splinted multi-unit coping	NSMU	0,99 ± 0,09	A,b	0,94 ± 0,14	A,d	1,02 ± 0,06	B,d

**Table 3:** *P* values of comparisons of impression techniques within each angulation model. *P* < 0.05 was accepted as statistically significant

	*P* values							
Model 1	Coronal Deviation	Angular Deviation	Model 2	Coronal Deviation	Angular Deviation	Model 3	Coronal Deviation	Angular Deviation
SH*NSH	***0.082***	0.032	SH*NSH	0.026	0.036	SH*NSH	***0.112***	0.013
SH*SNH	***0.087***	***0.066***	SH*SNH	0.031	0.022	SH*SNH	0.014	0.028
SH*NSNH	***0.062***	0.026	SH*NSNH	0.026	***0.097***	SH*NSNH	0.026	0.006
SH*SMU	0.011	***0.132***	SH*SMU	0.046	0.036	SH*SMU	0.047	0.043
SH*NSMU	0.016	0.012	SH*NSMU	***0.074***	0.037	SH*NSMU	0.047	0.022
NSH*SNH	***0.111***	0.031	NSH*SNH	0.018	0.044	NSH*SNH	0.026	0.009
NSH*NSNH	***0.117***	***0.172***	NSH*NSNH	0.042	***0.091***	NSH*NSNH	0.039	***0.096***
NSH*SMU	0.024	0.023	NSH*SMU	0.016	0.026	NSH*SMU	0.033	0.029
NSH*NSMU	0.031	***0.093***	NSH*NSMU	0.044	0.005	NSH*NSMU	0.045	0.037
SNH*NSNH	***0.091***	0.01	SNH*NSNH	0.043	0.037	SNH*NSNH	0.036	0.005
SNH*SMU	0.014	***0.072***	SNH*SMU	0.027	0.026	SNH*SMU	***0.119***	0.044
SNH*NSMU	0.018	0.022	SNH*NSMU	0.035	0.036	SNH*NSMU	0.045	0.047
NSNH*SMU	0.027	0.039	NSNH*SMU	***0.062***	0.041	NSNH*SMU	0.039	0.014
NSNH*NSMU	0.017	***0.132***	NSNH*NSMU	0.026	0.033	NSNH*NSMU	0.042	0.023
SMU*NSMU	***0.072***	0.026	SMU*NSMU	0.033	***0.185***	SMU*NSMU	0.025	***0.085***

**Table 4:** *P* values of comparisons of test models within each impression technique group. *P* < 0.05 was accepted as statistically significant

	*P* values											
	Coronal deviation						Angular deviation					
	SH	NSH	SNH	NSNH	SMU	NSMU	SH	NSH	SNH	NSNH	SMU	NSMU
Model 1–2	0.016	0.022	***0.099***	0.02	***0.095***	0.039	0.023	0.015	***0.127***	***0.159***	***0.069***	***0.745***
Model 1–3	0.044	0.012	***0.102***	0.019	***0.088***	0.042	0.036	0.036	***0.191***	0.018	***0.152***	0.041
Model 2–3	0.043	0.045	***0.736***	***0.223***	***0.123***	0.037	0.009	0.028	***0.166***	0.009	***0.084***	0.022

For model 1, abutment level impression techniques showed significantly higher coronal deviations than implant level techniques (*P* < 0.05). No statistically significant difference was detected among implant level impression techniques (*P* > 0.05). For model 2, the lowest coronal deviation values were found in the splinted non-hex coping (SNH) group; whereas, the highest values were detected in the non-splinted hexed coping (NSH) group (*P* < 0.05). For model 3, coronal deviations of splinted hexed coping (SH) and NSH groups were greater than the other groups (*P* < 0.05). SNH and splinted multi-unit coping (SMU) groups demonstrated the lowest deviation values (*P* < 0.05). When the effect of implant angulation was evaluated within each test group; SH, NSH, and non-splinted multi-unit coping (NSMU) groups showed significantly higher coronal deviation values with increased implant angulation (*P* < 0.05). Implant angulation did not affect the coronal deviations of SNH and SMU groups (*P* > 0.05) (Table 1).

For model 1, although splinted groups (SH, SNH, and SMU) revealed lower angular deviation values than non-splinted groups (*P* < 0.05), no significant difference was found among splinted groups (*P* > 0.05). For models 2 and 3, the lowest angular deviations were detected in SNH groups (*P* < 0.01). For both models, NSH and non-splinted non-hex coping (NSNH) groups demonstrated higher angular deviations (*P* < 0.05). Within-group evaluation revealed that increasing angular differences among copings did not affect the deviation values of SNH and SMU groups (*P* > 0.05).

## Discussion

In implant prosthodontics, transferring the intraoral positions of implants and abutments to the definitive casts as accurately as possible is of paramount important to achieve passive fitting prosthesis [7]. There are several studies focused on the factors that affect the precision of impressions. Also, there are reviews [7, 20, 23] concluded that the presence of multiple and angulated implants results in less accurate impressions. Different impression techniques, impression and splinting materials, impression copings have been evaluated; however, there is no consensus or clinical guideline regarding the impression protocol when multiple and angulated implants exist. Therefore, this study was conducted to determine the most accurate impression procedure for the aforementioned clinical situations. According to the statistical analysis, implant angulation; coping type and splinting impression copings had significant effects on the accuracy of impressions. Therefore, the null hypotheses were rejected.

Because of the nature of bone resorption in the anterior maxilla and anatomic limitations including maxillary sinus, parallel placement of implants is more challenging in the maxillary arch. In the anterior region, implants are often tilted labially and in posterior region, implants may be tilted parallel to the inclination of the maxillary sinus wall. In the present study, to better simulate the clinical conditions, maxillary models with anatomical undercuts were used. Also, the anterior implants were placed with a labial inclination and the posterior implants were placed with a distal inclination. Thereby, angulation differences created among impression copings. In most of the previous studies [6, 19, 24], trays were removed perpendicular to the horizontal plane. A special appliance was used in this study to simulate the strain during the tray removal, more effectively. With this appliance, removal force was applied only in two directions as similar as to the procedure in the mouth and the force was standardized by using the pulleys.

Polyether and vinyl poly-siloxane have been suggested as materials of choice for implant impression procedures [7, 20]. Although rigidity and dimensional stability of polyether has been reported to provide accurate implant impressions in fully edentulous and multi-implant cases [4], the use of a material with higher elastic recovery, as vinyl poly-siloxane, would be more suitable for easy removal of the tray. With this manner, the permanent deformation caused by the stress between impression copings and the material may be reduced, especially in the case of nonparallel and internal connection implants [4, 8, 25]. Accordingly, mono-phase vinyl poly-siloxane impression material was preferred in this study.

Although there are numerous studies [15, 26–30] in the literature that examined the effect of splinting and non-splinting the impression copings; the results are inconsistent and none of the two procedures was reported to be superior [9, 17, 20, 23]. Auto-polymerizing acrylic resin is the most frequently evaluated splinting material; however, polymerization shrinkage is the main problem that reduces the accuracy of resin. Hence, sectioning and re-splinting of the acrylic resin bars are recommended to minimize this shrinkage [23, 31]. Lowest shrinkage values were reported after 24 h of polymerization [32] and therefore, in the present study, auto-polymerizing acrylic resin was applied using dental floss, sectioned after 24 h and reconnected before impression procedure.

In internal connection implants, the length of the implant and impression coping connection have been reported to influence the accuracy of multiple and angulated implant impressions [4, 16]. Although no significant difference was found among the coronal deviations of implant level impression techniques in parallel copings; significantly lower deviation values were calculated in non-hex coping groups (SNH and NSNH) and in splinted multi-unit coping group of angulated models. Better accuracy in non-hex coping groups can be related to the 2 mm shorter connection area of copings that may reduce the removal stress caused by implant divergence. In comparison to implant level impressions with hexed impression copings, abutment level impressions with multi-unit abutments showed lower coronal deviations when multi-unit copings were splinted (Table 1). This finding is in agreement with a previous study [24] that reported higher accuracy of abutment level impressions in the case of distally tilted implants. Since the multi-unit copings are not engaging to abutments and the geometry of the coping allows rotation; splinting the multi-unit copings would provide better precision of impressions.

Splinting the impression copings of parallel implants demonstrated significantly lower angular deviations regardless of impression level or coping type (Table 2). Previous studies [33, 34] suggested that splinting the copings did not affect the accuracy of impression when the implants are parallel to each other. However, in those studies, trays were removed perpendicular to the occlusal plane which did not simulate the clinical conditions. Simulating the tray removal path in the current study might affect the results in non-splinted groups even the copings were not angulated. Significantly, lowest angular deviations were determined in SNH groups of both angulated models, followed by abutment-level impression groups (SMU and NSMU).

In angulated models, most anterior and posterior implants were inclined buccally and distally; hence, a total of 20- and 40-degrees of divergence occurred in the sagittal plane in models 2 and 3, respectively. When the effect of angulation was assessed within each test group, coronal and angular deviations of hexed coping groups increased as the angular differences increased. However, coronal and angular deviations of splinted non-hex and splinted multi-unit groups were not influenced by the angulation of the implants. These findings may be attributed to the shorter connection area of non-hex copings and also, conical and external connection of multi-unit abutments which eliminates the increased contact area between the implant and coping. This connection design may reduce the movement of copings during the removal of the tray [20]. Therefore, using non-engaging (non-hex and multi-unit) copings and splinting them with a rigid material can be recommended to obtain more accurate impressions in the case of multiple and angulated implants.

Inconsistent results reported in the literature may be explained by different methodologies to assess the accuracy of impressions. In most of the studies, the precision of impression was evaluated by measuring the distances between implants and by calculating the positional changes of the analogues in duplicate casts with the aid of either coordinate measuring machine [22, 24, 35]; measuring microscope [36]; profile projector [4, 25] or standardized photographs [37]. In the present study, a digital method described previously [6, 8] was preferred to obtain more precise results with 3-dimensional analyses. Although significant differences were found among the different test groups evaluated; mean coronal deviations detected in this study were in a range of 0.33–0.76 mm (33–76 μm) (Table 1). These deviations were higher than the values reported in previous studies [6, 8] that used the same assessment method. Higher deviations can be explained with the higher number of implants used and greater angulation difference composed among implants in the current study. It has been suggested that in a good impression, a discrepancy of 50 μm can be detected in any axis [28]. Therefore, from a clinical point of view, when all the deviation values are taken into consideration, for both parallel and angulated situations, the use of non-hex copings with splinted open tray technique can be recommended. On the other hand, machining tolerance of implant components ranged from 22 to 100 μm [38] may also be an important factor that affects the deviation values [7].

Although all impression steps were performed by a single clinician to standardize the procedures, reproducibility of the impression within the clinician was not evaluated which can be considered as a limitation. Other possible limitations of the current study were that the axial rotations of the copings were not detected, the implants were inclined only on the sagittal plane, and different depths of implant insertion were not evaluated. Furthermore, impressions were made under ideal conditions without the effect of soft tissues or saliva. In future studies, to achieve more clinically relevant results, it may be recommended to evaluate the passive fit of prosthesis fabricated on the casts obtained from different impression procedures.

Due to the improvements in digital dentistry, digital implant impressions using intraoral scanners became popular. Intraoral scanning has been reported as a time efficient and comfortable way of impression-making preferred by the patients and as a technique which shows similar accuracy in comparison to the conventional techniques for single implants or partially edentulous cases. The types of scanbodies and intraoral scanners also affect the accuracy of digital impressions [39]. When multiple implants are available in complete edentulism, the length of the span becomes longer due to the inter-implant distance and the number of images that the scanner needs to capture increases which results in higher cumulative errors and thereby less accuracy [40, 41]. As the future of dentistry is going towards digital manner, researches regarding implant impressions should focus on digital impression and trueness of different intraoral scanners. Since the data regarding the accuracy of intraoral scanning is limited in the case of multiple and angulated implants, and distortion risk of impression material due to angulation difference is eliminated with the aid of intraoral scanning, in further studies, conventional and digital impression techniques should also be compared in angulated implants.

## Conclusions

Within the limitations of this in vitro study, the following conclusions can be drawn:

1. Implant angulation, impression coping type, and splinting the impression copings had significant effects on the accuracy of impressions.
2. In parallel implants, lower coronal angulations were detected in implant level impressions, and lower angular deviations were found in splinted groups.
3. For angulated models, the most accurate impressions were obtained from splinted non-hex coping group.
4. Hexed copings demonstrated significantly higher deviations in angulated models.

## Data Availability

Not applicable.
